# Joint effects of population size and isolation on genetic erosion in fragmented populations: finding fragmentation thresholds for management

**DOI:** 10.1111/eva.12154

**Published:** 2014-03-20

**Authors:** María Méndez, Matthias Vögeli, José L Tella, José A Godoy

**Affiliations:** 1 Estación Biológica de Doñana (EBD-CSIC)Sevilla, Spain; 2 Department of Biology, University of SaskatchewanSaskatoon, SK, Canada; * Schulstrasse 47, 5423, Freienwil, Switzerland

**Keywords:** conservation genetics, genetic diversity, habitat fragmentation, inbreeding, population genetics

## Abstract

Size and isolation of local populations are main parameters of interest when assessing the genetic consequences of habitat fragmentation. However, their relative influence on the genetic erosion of local populations remains unclear. In this study, we first analysed how size and isolation of habitat patches influence the genetic variation of local populations of the Dupont's lark (*Chersophilus duponti*), an endangered songbird. An information-theoretic approach to model selection allowed us to address the importance of interactions between habitat variables, an aspect seldom considered in fragmentation studies, but which explained up to 65% of the variance in genetic parameters. Genetic diversity and inbreeding were influenced by the size of local populations depending on their degree of isolation, and genetic differentiation was positively related to isolation. We then identified a minimum local population of 19 male territories and a maximum distance of 30 km to the nearest population as thresholds from which genetic erosion becomes apparent. Our results alert on possibly misleading conclusions and suboptimal management recommendations when only additive effects are taken into account and encourage the use of most explanatory but easy-to-measure variables for the evaluation of genetic risks in conservation programmes.

## Introduction

Human activities are threatening a large number of species worldwide through habitat loss and fragmentation (Andren 1994). Changes in land use often reduce the size of populations and increase their isolation to limits where an increased susceptibility to stochastic factors may precipitate their extinction (Lande 1993; Hanski and Ovaskainen 2000). Loss of genetic diversity, accumulation of genetic load and increased rates of inbreeding may reduce birth and increase death rates in small populations, thereby reducing fitness (Jaquiery et al. 2009). This effect – usually referred to as inbreeding depression – together with the loss of adaptive potential, has shown to significantly increase extinction probabilities, in both simulation (Saccheri et al. 1998; Tanaka 2000; O'Grady et al. 2006) and empirical studies (Saccheri et al. 1998; Vilas et al. 2006). Due to the critical importance for species persistence and evolution, genetic diversity has been identified by the International Union for Conservation Nature (IUCN) as one of three levels of priority for global conservation of biodiversity (McNeely et al. 1990). Genetic monitoring should thus become a critical ingredient of conservation and management plans (Laikre et al. 2009).

Two main microevolutionary processes influence genetic patterns in declining and fragmented populations: genetic drift and gene flow. These processes are determined by local population size and interpatch connectivity, parameters that are commonly taken into account in conservation planning under a metapopulation theory framework (Hanski 1999). While genetic drift causes random fluctuations of allelic frequencies and loss of genetic diversity through time as a function of effective population size, dispersal-mediated gene flow can buffer these effects in local populations. The outcome of these two processes is predicted under simplified equilibrium models and pure-drift models. This is, however, not the case under the nonequilibrium situation imposed by the recent or on-going fragmentation of wildlife populations, where gene flow and local drift may vary over time and among patches. Nevertheless, larger and more connected populations often maintain higher genetic diversity and lower inbreeding and differentiation than smaller and more isolated populations (Frankham 1996; Cruzan 2001; Jacquemyn et al. 2009). However, the effects of population size and isolation are usually considered independently (e.g. Prentice et al. 2006; Telles et al. 2007; Dillane et al. 2008; Meeuwig et al. 2010), and only a few empirical studies have investigated the joint effects of both parameters and their interaction on the genetic dynamics of wild populations (Gonzalez-Varo et al. 2010; Lange et al. 2010; Wang et al. 2010). Contrary to theoretical expectations, these studies did not find this interaction to be a major factor affecting genetic patterns.

Critical thresholds in habitat fragmentation have been defined as an abrupt and nonlinear change in some parameter across a small range of habitat loss (With and King 1999). Despite of empirical evidence for critical thresholds in habitat fragmentation (e.g. Swift and Hannon 2010), they are rarely addressed in studies of genetic variability, inbreeding or differentiation (Cruzan 2001; Lowe et al. 2005; Ezard and Travis 2006; Bruggeman et al. 2010; Lange et al. 2010). A better understanding of drift–gene flow interactions and fragmentation thresholds for genetic erosion is thus crucial to determine how human-induced habitat fragmentation can affect population viability and long-term evolutionary processes through their impact on genetic variation. Furthermore, the identification of critical thresholds should contribute to more efficient conservation in fragmented landscapes by helping to gauge the relative benefit of acting on population size or connectivity for the maximization of the short-term genetic viability of local populations.

In this study, we evaluated the effects of population size, isolation and their interaction on the genetic patterns (diversity, inbreeding, relatedness and differentiation) of recently fragmented populations. Furthermore, we aimed to identify the thresholds of population size and isolation beyond which genetic erosion starts to accumulate to detectable levels. We focused on the endangered Dupont's lark (*Chersophilus duponti*), a markedly sedentary steppe specialist passerine suffering from habitat loss and fragmentation in recent decades (Tella et al. 2005). Capture–mark–recapture methods have only detected reduced, short-distance movements between local populations albeit being separated only by few kilometres (Laiolo et al. 2008; Vogeli et al. 2008). The high isolation of occupied habitat remnants and the species' low dispersal propensity have led to a loss of genetic diversity and to an increase in genetic structure (Mendez et al. 2011). Hence, the life history of our study species and its spatially structured habitat provide a good scenario for studying the consequences of anthropogenic fragmentation on population genetics and viability. We focus in particular on those landscape and population variables that are easy to monitor in the field and are frequently used in management. By doing so, we aim to increase the relevance of this study for conservation practice and to facilitate the application of this approach to other species that are threatened by recent or ongoing fragmentation.

## Materials and methods

### Study species and sampling

**Figure 1:**
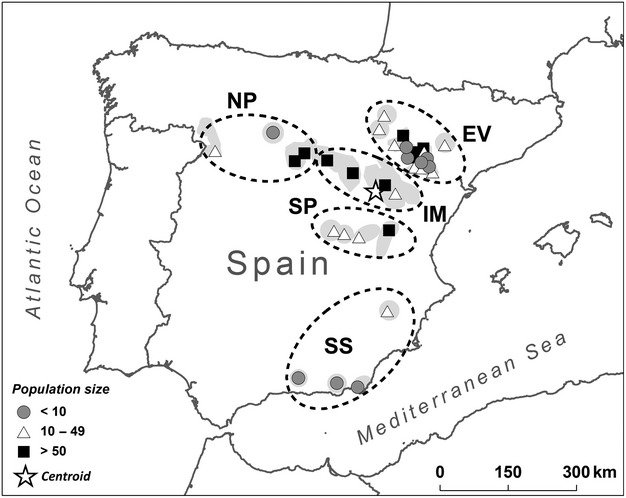
Location of the sampled Dupont's lark local populations in Spain. Different symbols indicate different population sizes (number of territorial males). Dark grey areas show the approximate distribution of the Dupont's lark in Spain based on Suárez 2010 (discontinuities among neighbouring small patches are not shown at this scale). The centroid indicates the mean geographical centre of all populations. Dashed ellipses show the topographical areas identified by Laiolo and Tella (2006a): EV, Ebro Valley; NP, Northern Plateau; SP, Southern Plateau; IM, Iberian Mountains; SS, Southern Spain.

The Dupont's lark is an endangered songbird whose habitat is highly restricted to steppe areas with natural vegetation in Spain and North Africa (Cramp 1988). The Spanish population has been confined to a series of fragments of variable size and degree of isolation, which collectively may hold as few as 2200–2700 breeding territories defended by males (Suárez 2010). The number of breeding pairs may be much smaller given a high male-biased adult sex-ratio in this species (Tella et al. 2004). Habitat loss and fragmentation have been sufficient to extirpate many local populations since the 1980s (Tella et al. 2005; Suárez 2010; Vogeli et al. 2010) and to noticeably alter cultural transmission, demographic and genetic patterns of the species at both local and regional scales (Laiolo and Tella 2005, 2006a, 2007; Laiolo et al. 2008; Méndez et al. 2011; Vogeli et al. 2011). This process has not occurred homogeneously across the species distribution, being especially intense in the peripheral topographical areas defined by Laiolo and Tella (2006a; Fig. 1). The suitable habitat for Dupont's lark in the core of the distribution [Iberian Mountains and Ebro Valley (EV)] has remained more connected, while habitat loss has been more pronounced in the periphery of the species' distribution in recent decades (Northern Plateau, Southern Spain and Southern Plateau; Laiolo and Tella 2006a,b). In this sense, Dupont's lark populations are suffering a centripetal process of contraction and isolation following recent fragmentation.

During spring to autumn of 2002–2008, we attracted birds using playback and employed clap nets baited with meal worms to capture 506 adults of Dupont's larks in 36 localities, covering the whole distribution range of the species in Spain (Fig. 1). Ninety-three per cent of sampled individuals were males, as determined by molecular analyses (Vogeli et al. 2007). We considered each steppe patch as the sampling unit, so areas with more patches, as the EV, have more sampling points (Fig. 1). A drop of blood was extracted for molecular analysis and stored in pure ethanol. All birds were released at the site of capture, and handling was specifically approved by all competing wildlife agencies and administrations (further details are provided in previous studies: Vogeli et al. 2007). This research complied with the norms of the Spanish Animal Protection Regulation, RD1201/2005, about protection of animals used in scientific research, which conforms to European Union Regulation 2003/65.

### Genetic analyses

DNA was extracted from blood samples following the protocol described in Gemmell and Akiyama (1996) with two steps of chloroform. All samples were amplified with 14 species-specific microsatellite loci (Mendez et al. 2011). None of the microsatellite markers showed evidence for Hardy–Weinberg disequilibrium, allelic dropout or null alleles. We estimated expected heterozygosity (*H*_e_) with GenePop on the Web (Raymond and Rousset 1995; Rousset 2008) and allelic richness (*A*_R_) using the rarefaction method to correct for unequal sample sizes with Fstat 3.9.3 (Goudet 1995). We computed the population inbreeding coefficient (*F*_IS_) and the average pairwise relatedness estimator (*R*). *F*_IS_ was calculated with Genetix (Belkhir 1997) and reflects deviation of Hardy–Weinberg equilibrium due to nonrandom mating within populations. *R* was calculated with GeneAlEx 6 (Peakall and Smouse 2006) using allelic frequencies of the global population. Moreover, based on previous studies (Mendez et al. 2011), we calculated a population differentiation index under a pure-drift model (*F*) with 2MOD (Ciofi et al. 1999), which reflect the accumulated inbreeding in a population by genetic drift. Finally, we estimated population-specific genetic differentiation through the arithmetic mean of the population pairwise *F*_ST_ (Weir and Cockerham 1984), 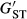 (Hedrick 2005) and *D*_ST_ (Jost 2008) values with Genetix and the Software for the Measurement of Genetic Diversity (SMOGD version 1.2.5; Crawford 2010).

### Size and isolation of populations

Three of the 36 surveyed localities held a small number of sampled individuals (*n* < 10). They were merged with their closest neighbour locality to increase sample sizes for analyses, yielding 33 local populations (Méndez et al. 2011). All three merged pairs of localities were in the core of the distribution, separated from each other by 1–4 km (less than one-fifth of the maximum dispersal distance detected in the study area, Laiolo et al. 2007) and interconnected by suitable habitat. Moreover, pairs of merged localities did not show signs of genetic differentiation (low and nonsignificant pairwise *F*_ST_) and were grouped in the same genetic cluster (Méndez et al. 2011). Dupont's lark population sizes were characterized by two different estimates: (i) the number of male territories (N.pop), estimated from vocalizations in previous studies (Suárez 2010; Vogeli et al. 2010), and (ii) the patch size (area) of each population. Area was calculated as the surface of natural steppe vegetation occupied by the Dupont's lark, obtained from land-use maps and aerial orthophotographs (Vogeli et al. 2010). Both estimates are significantly correlated (linear regression, *R*^2^* *=* *0.616, *P *<* *0.001, *N* = 33), and both are generally good indicators of population sizes (Laiolo et al. 2008). They can differ, however, in small patches where the reduction in patch size causes an increase in Dupont's lark densities (‘crowding effect’), thus impeding a stronger relationship between patch size and population size (Laiolo and Tella 2006a; Vogeli et al. 2010; Méndez et al. 2011). Besides, they also differ in the time and costs associated to data collection. N.pop estimates need exhaustive field work for obtaining the number of territorial males (Tella et al. 2005). Dupont's lark is a very elusive, secretive and difficult to observe terrestrial species, to the point that its occurrence and distribution in Spain was not roughly assessed until the late 1980s (Garza and Suárez 1990). Thus, mapping territorial males through their vocal activity is the only way to properly estimate local population sizes (Tella et al. 2005; Laiolo and Tella 2006a). Calculating area is, however, relatively easy, because areas of natural steppe vegetation adequate for the study species are island-like remnants in a landscape dominated by agriculture, so they are easily identified with basic GIS procedures (Vogeli et al. 2010).

We calculated population isolation by the two approximations that were identified in a previous study (Vogeli et al. 2010) as the best predictors of Dupont's lark occurrence at the metapopulation scale: the Euclidean distance from each population to its nearest neighbour population (D.near), and the distance from each population to the average coordinates of all Spanish Dupont's lark populations (D.centre). While the former is straightforward to estimate, the latter requires a complete knowledge of the distribution of the species and the application of GIS tools, so it could be more difficult to incorporate in management practice. Geographical patch centres were used for calculating both isolation indexes.

### Statistical analysis

We used generalized linear models to assess the relationships between the population-based genetic indexes (*H*_e_, *A*_R_, *F*_IS_, *R*, *F*, *F*_ST_, 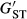, *D*_ST_) and ecological and demographic attributes of each local population (i.e. population size and isolation). Population size (characterized by N.pop and area) and isolation (calculated as D.centre and D.near) were fitted as explanatory variables. Following our hypotheses, we designed *a priori* five alternative models to assess the relationships between the explanatory variables and the eight response variables. The first three univariate models only included single explanatory variables (N.pop, area, D.centre or D.near) to assess their effects separately. The fourth model included area and isolation, while the fifth included area, isolation and the interaction between area and isolation. All models were tested twice alternatively using D.near and D.centre as isolation measures. We followed a model selection strategy using an information-theoretic approach (Johnson and Omland 2004), computing the Akaike information criterion corrected for small sample sizes (AIC_c_) and relative weight of evidence for each model (*w*_*i*_) as the probability of model *i* being the best model for the observed data, given the set of candidate models. The most parsimonious model for each genetic index was selected based on a lower AIC_c_ and higher *w*_*i*_ (Johnson and Omland 2004). All statistical analyses were performed in R 2.11.1 software (R Development Core Team 2011). Models were built with a normal distribution of errors and the identity link function. N.pop, area and D.near were log-transformed to attain normality. There was no evidence of overdispersion, and residuals fitted to a normal distribution, indicating that distributions and error structures were appropriate (Rushton et al. 2004). We estimated parameters and plotted the finally selected, most parsimonious models only if the retained explanatory variables reached statistical significance at *P *<* *0.05. Due to the spatial distribution of the populations, we analysed whether spatial autocorrelation might impact the planned analysis. We consequently assessed final model residuals through Moran's *I* correlograms (Dormann et al. 2007). Spatial autocorrelation of residuals was only significant in models relating *H*_e_, *F*_IS_ and *F* to N.pop and D.near, indicating they did not adequately capture the spatial structure of the genetic variance. Finally, we performed piecewise and exponential regression models to detect nonlinear thresholds in final models with only one independent variable (Toms and Lesperance 2003; Laiolo et al. 2008). Following AIC_c_, piecewise models performed better in all cases, so we only use those for detecting thresholds.

## Results

**Figure 2:**
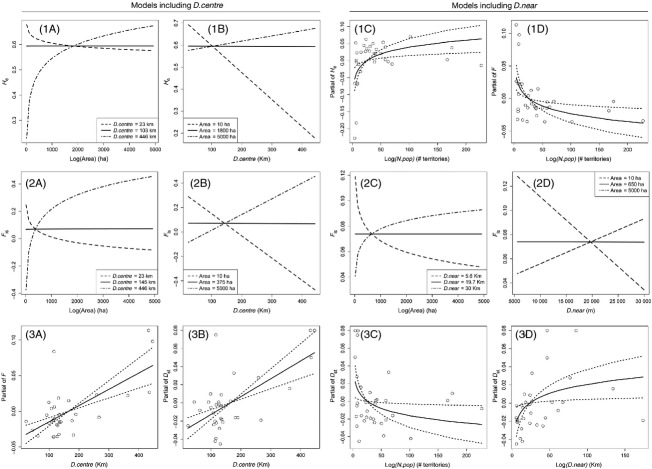
Predicted relationships and partial contributions of population and ecological variables on genetic indexes: patch size (*area*), population size as number of male territories (*N.pop*), distance to the centroid of all Spanish populations (*D.centre*) and distance to the nearest population (*D.near*).

Our exhaustive sampling of the whole Spanish (and thus European) Dupont's lark distribution captured a wide range of both the genetic (*H*_e_ and *A*_R_, *F*_IS_, *R*, *F*, *F*_ST_, 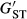 and *D*_ST_) and the ecological (area, N.pop, D.near and D.Centre) conditions (Table S1). Top ranking models for *A*_R_, *R* and *F*_ST_ and 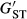 were similar to those for *H*_e_, *F*_IS_ and *D*_ST_. For simplicity, we include the formers in Supporting Information and report the latters and *F* in Tables 1 and2 and Fig. 2. Selected models included size or isolation variables or their interaction (Table 1) and explained between 13.6% (*D*_ST_) and 65.3% (*H*_e_) of the variation (Adj. *R*^2^) in the genetic parameters (Table 2).

**Table 1:** Evaluation of alternative models for genetic parameters based on population size and isolation variables.

	Models with *D.centre*					Models with *D.near*				
	*N.pop*	*Area*	*D.centre*	*Area + D.centre*	Area^*^D.centre	*N.pop*	*Area*	*D.near*	*Area + D.near*	Area^*^D.near
*H*_e_										
AIC_c_	−91.9	−85.9	−99.6	−103.6	−**115.6**	−**91.9**	−85.9	−87.3	−89.8	−89.1
ΔAIC_c_	23.8	29.7	16.0	12.0	**0.0**	**0.0**	5.9	4.5	2.1	2.7
*w*_*i*_	0.0	0.0	0.0	0.0	**1.0**	**0.6**	0.0	0.1	0.2	0.1
*F*_IS_										
AIC_c_	−63.2	−64.2	−63.0	−61.8	−**79.0**	−63.2	−64.2	−63.2	−62.1	−**69.2**
ΔAIC_c_	15.7	14.8	16.0	17.2	**0.0**	6.0	5.0	6.0	7.1	**0.0**
*w*_*i*_	0.0	0.0	0.0	0.0	**1.0**	0.0	0.1	0.0	0.0	**0.8**
*F*										
AIC_c_	−123.0	−119.5	−**139.5**	−137.1	−134.6	−**123.0**	−119.5	−121.9	−120.0	−117.0
ΔAIC_c_	9.5	20.0	**0.0**	2.4	4.9	**0.0**	10.5	8.1	10.5	13.0
*w*_*i*_	0.0	0.0	**0.7**	0.2	0.1	**1.0**	0.0	0.0	0.0	0.0
*D*_ST_										
AIC_c_	−133.2	−127.4	−**145.8**	−143.4	−141.1	−**133.6**	−127.4	−**133.4**	−131.0	−129.0
ΔAIC_c_	12.6	18.5	**0.0**	2.4	4.7	**0.2**	6.0	**0.0**	2.4	4.4
*w*_*i*_	0.0	0.0	**0.7**	0.2	0.1	**0.4**	0.0	**0.4**	0.1	0.0

Genetic parameters: *H*_e_ = expected heterozygosity, inbreeding (*F*_IS_), accumulated inbreeding (*F*) and differentiation (*D*_ST_). Model selection repeated for two alternative measures of isolation: distance to the centroid of all populations (*D.centre*) and distance to the nearest population (*D.near*). *N.pop* = male territories, *Area* = patch size. Additive (+) and interactive (^*^) effects were considered. The corrected Akaike information criterion (AIC_c_), difference with the best model (ΔAIC_c_) and the relative weight of evidence for each model (*w*_*i*_) are reported (two equally good models when *w*_*i*_ < 0.5). Statistics of the most parsimonious model are highlighted in bold.

**Table 2:** Generalized linear models. Model coefficients are shown separately for distance to the centroid of all populations (*D.centre*) and distance to the nearest population (*D.near*).

	Final models with *D.centre*						Final models with *D.near*					
	Adj. *R*^2^		*Intercept*	*Area*	*D.centre*	*Area* × *D.centre*	*Adj*. *R*^2^	*Intercept*	*Area*	*D.near*	*Area* × *D.near*	*N.pop*
*H*_e_	0.653	Estimate	0.776**	−0.024*	−0.002**	2 × 10^−4^**	0.225	0.472**				0.029*
		SE	0.065	0.011	3 × 10^−4^	6 × 10^−5^		0.031				0.009
*F*_IS_	0.417	Estimate	0.495**	−0.072**	−0.003**	5 × 10^−4^**	0.215	2.300*	−0.365*	−0.228*	0.037*	
		SE	0.114	0.019	6 × 10^−4^	1 × 10^−4^		0.719	0.120	0.071	0.011	
*F*	0.436	Estimate	0.019*		2 × 10^−4^**		0.248	0.113**				−0.017*
		SE	0.009		4 × 10^−5^			0.017				0.005
*D*_ST_	0.410	Estimate	0.084**		2 × 10^−4^**		0.136	0.155**				−0.012*
		SE	0.008		4 × 10^−5^			0.017				0.005
		Estimate (2)					0.141	−0.028		0.014*		
		SE (2)						0.058		0.006		

Significant coefficient estimates:

* *P* < 0.05;

** *P* < 0.001.

Population size (N.pop) was retained as a statistically significant predictor in final models when using D.near as isolation measure and explained 22% of the variance in heterozygosity (*H*_e_) and 24% of the variance in accumulated inbreeding (*F*) (Table 2). Moreover, N.pop was also retained in the best competing model for *D*_ST_ (14% of the variance explained; Table 2). Genetic diversity increased logarithmically, whereas genetic differentiation decreased logarithmically with population size (Fig. 2; panels 1B, 3B). When analysing these relationships with piecewise regression models, we found a significant breakpoint at 19 male territories in the case of *H*_e_ (Adj. *R*^2^* *=* *0.40, *F*_4,33_* *=* *8.10, *P* < 0.001) and *F* (Adj. *R*^2^* *=* *0.25, *F*_4,33_* *=* *4.52, *P* < 0.05) and at 16 for *D*_ST_ (Adj. *R*^2^* *=* *0.21, *F*_4,33_* *=* *3.92, *P* < 0.001). Therefore, populations with <16–19 males were prone to the loss of genetic diversity and to the accumulation of inbreeding and differentiation.

Patch size (area) was not included as a single predictor but played a key role in several final models through its interaction with isolation (see below).

The distance to the centroid of all populations (D.centre) performed better as an isolation measure than the distance to the nearest population (D.near) for most genetic parameters (Table 2). D.centre was selected in all the genetic differentiation models and the accumulation of inbreeding model, explaining 25%, 40%, 41% and 43% of the variance of *F*_ST_, 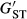, *D*_ST_ and *F*, respectively. On the other hand, D.near was selected instead of D.centre for *A*_R_, *F*_ST_, *D*_ST_ and 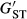, performing similarly to N.pop (Table 1 and Supporting Information). In summary, genetic differentiation and the accumulation of inbreeding increased with isolation irrespectively of the isolation measure used (Fig. 2, panels 3A and 4A). The piecewise regression model showed a significant breakpoint for *D*_ST_ in 30 km to the nearest population (Adj. *R*^2^* *=* *0.10, *F*_4,33_* *=* *2.16, *P* < 0.001).

Models including the interaction between patch size and isolation explained the highest percentages of the observed variance in genetic diversity (*H*_e_ and *A*_R_), inbreeding (*F*_IS_) and relatedness (*R*) (65%, 55%, 42% and 24%, respectively; Table 2 and Supporting Information). Indeed, this interaction was retained in the most parsimonious models explaining inbreeding and relatedness, regardless of the isolation metric used. Figure 2 illustrates size and isolation interactions for *H*_e_ (panel 1A) and *F*_IS_ (panel 2A,B). In all cases, genetic indexes varied differently with patch size depending on their degree of isolation, or conversely, they responded differently to an increase in isolation depending on their size. To better understand these interactions, we plotted the lowest and highest values of isolation found in our data, and the one where the effect of area did not promote a change in genetic indexes. Above or below this level of isolation, the relationships between genetic indexes and patch area changed, either increasing or decreasing. With low values of isolation (23 km to the centroid or 5.6 km to the nearest population), genetic diversity and inbreeding or relatedness decreased with patch size. Distances of 103–145 km to the centroid of all populations or at 19 km to the nearest population resulted in stable genetic indexes in all patch sizes. At the same time, patch sizes of 300, 375, 650 and 1800 ha made genetic indexes independent of their degree of isolation (intersection for *R*, *F*_IS_ with D.centre and D.near and *H*_e_, respectively). Finally, larger values of isolation resulted in increasing genetic diversity and inbreeding with patch size.

## Discussion

Our exhaustive sampling of fragmented Dupont's lark populations covering the whole European distribution and almost the 25% of the estimated male's territories, and the use of model selection techniques, allowed us to evaluate the relative importance of population (or patch) size and isolation in explaining the observed variance in genetic parameters related to diversity, inbreeding and differentiation.

**Figure 3:**
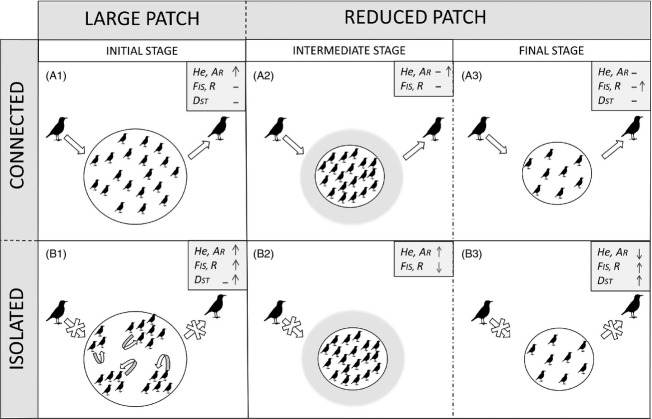
General overview of the genetic consequences of patch size reduction and isolation on Dupont's lark population genetics. Based on this study, genetic diversity and inbreeding of Dupont's lark populations change depending of the size and isolation of steppes patches. After fragmentation, (panel A) connected and reduced patches under 1800 or 300 ha reduce their genetic diversity and increase their inbreeding, while (panel B) isolated (more than 13–19 km to the nearest population) and reduced patches first increase their genetic diversity and reduce their inbreeding and finally reduce their diversity and increase their inbreeding. We found that 16–19 males territories and 30 km to the nearest population are the fragmentation thresholds needed to support the initial genetic conditions. Arrows indicate migration into or from local populations at initial, intermediate and final stages of patch size reduction and isolation. Shadow areas around circles indicate recent patch contractions. The number and distribution of birds within populations illustrate changes in population size and density throughout the stages of fragmentation. Symbols ↑, ↓ and – indicate increase, decrease or no change, respectively, in the corresponding genetic index: genetic diversity (*H*_e_), Alelic richness (*A*_R_), inbreeding (*F*_IS_), relatedness (*R*) or differentiation (*D*_ST_).

Population size had a major influence on genetic indexes of Dupont's larks: genetic diversity increased and differentiation decreased with local population size, being the expected outcome of a less intense genetic drift in larger populations (Fig. 3, panel A). Nevertheless, this effect is likely to vary across the range of population sizes, due to the nonlinear relationship of these variables, and through time. Time is an important variable that could not be included directly in our analyses, but could be indirectly captured by one of our isolation variables (D.centre, see below). Dupont's lark populations holding <19 male territories have lost genetic diversity and increase the accumulated inbreeding and above 16 have accumulated differentiation to detectable levels. Below these population size thresholds, genetic erosion was minor or undetectable (Fig. 3-A.3/B.3).

The other parameter of interest, isolation, proved also of great importance for another genetic variable: genetic differentiation increased with isolation (Fig. 3, panel B). As predicted by theory, gene flow between habitat patches may buffer the effects of genetic drift. In this sense, the effects of isolation are expected to be stronger and more detectable in species with low dispersal capability (Segelbacher et al. 2010) than in more dispersive species (e.g. Lindsay et al. 2008; Canales-Delgadillo et al. 2012), but also in those populations with small populations sizes suffering more intense genetic drift (Frankham et al. 2002). Dupont's lark presents both of these two characteristics: low dispersal capability and small populations sizes (Laiolo et al. 2007; Vogeli et al. 2010). In this study, isolation of local populations was measured either as the linear distance to the nearest population or as the distance to the centroid of all populations. While both measures are meant to quantify the degree of population isolation, they likely do so at different spatial and temporal scales. On the one hand, the distance to the nearest population reflects more contemporary chances in dispersal among patches. In this sense, we found that genetic differentiation increased faster at distances above 30 km to the nearest population than under this threshold. On the other hand, the distance to the centroid of all populations performed better in reflecting long-term population dynamics, that is, it may better capture the stronger effect of genetic drift expected in edge populations due to their longer time since isolation or to a more intense population size reduction and isolation. This latter variable is critically important, but usually missing from explanatory models due to the difficulty in obtaining direct measures (see Kyle and Strobeck 2002; Vucetich and Waite 2003; Gapare and Aitken 2005).

Most importantly, the interaction between patch size and isolation explained a much higher proportion of the variance in genetic diversity, inbreeding and relatedness than each of these variables alone, or when only additive effects were considered. Although the product of population size by isolation (captured in genetic models by the migration rate) is considered determinant for the spatial variance in gene frequency in population genetics theory, only a few empirical studies of wild populations have explicitly evaluated this interactive effect. Some studies considered fragmentation as the product of size and isolation, making it impossible to disentangle the relative effects of these interacting variables (Lange et al. 2010). Others included the interaction between size and isolation in the process of model selection, yet it was not retained during the model selection process (Wang et al. 2010). To our knowledge, this is the first study showing statistically significant effects of this interaction on the genetic response of wild populations to fragmentation, and its higher explanatory power with respect to the single and additive effects.

### Interactive effects of patch size and isolation on genetic patterns

As the effect of patch size on genetic patterns varied depending on the isolation of the patch, we discuss here our model predictions according to three basic scenarios of isolation.

In the first scenario, where isolation is high enough to hamper gene flow, genetic diversity, inbreeding and relatedness of the Dupont's lark increased in local populations with patch size (Fig. 3, panel B). In line with our predictions, larger patches held more male territories (and thus more individuals) and thus retained more genetic diversity over time (Fig. 3-B.1). The increase in relatedness and inbreeding with patch size, however, is not as straightforward to explain. Dispersion is constrained in highly isolated populations (Gonzalez-Varo et al. 2010) and for our model species in particular, which shows extremely low propensity for dispersal (Laiolo et al. 2007). Hence, individuals may try to remain in their natal patches despite the negative effects for the dynamics of the population (Delgado et al. 2011). This tendency may be strengthened by conspecific attraction in our study species, which may also result in high levels of relatedness and inbreeding, and increased opportunities for spatially structured physiological, genetic and cultural patterns within patches (Laiolo and Tella 2005, 2008; Méndez et al. 2011; Fairhurst et al. 2013). Therefore, some of the increase in *F*_IS_ with population size might be due to the sampling across genetically differentiated subpopulations (i.e. Wahlund effect) in larger and less dense patches when these are highly isolated. These populations might therefore favour the maintenance of overall genetic diversity through the formation of spatially segregated groups of related individuals, although inbreeding and average relatedness will increase. Conversely, small isolated populations show less genetic diversity due to the combination of a lower effective population size and, probably, a longer time since isolation (Fig. 3-B.3). Surprisingly, inbreeding and relatedness are lower in recently contracted and isolated populations, which could be due to the transient high densities occurring in these populations (‘Crowding effect’; Laiolo and Tella 2006a; Vogeli et al. 2010; Méndez et al. 2011) favouring the contact among genetically differentiated groups within the patch.

In a second scenario, we focus on populations with intermediate levels of isolation and higher levels of gene flow. This should theoretically favour genetic diversity and reduce inbreeding and relatedness. In this sense, inbreeding and relatedness in Dupont's larks populations decreased with patch size (Fig. 3-A.1). Intriguingly, we detected a slightly increase in genetic diversity and inbreeding in well-connected populations with smaller patch sizes (Fig. 3-A.2/A.3). This effect can be due either to a statistical artefact due to the relatively small sample sizes, or to the fact that those smaller core populations may reflect a prefragmentation situation in which the occurrence of spatial genetic groups in larger patches results in heterozygote deficiency (due to intrapatch spatial structure), high overall diversity and some inbreeding (Fig. 3-B.1).

The third scenario depicts populations where varying patch sizes do not cause relevant changes in genetic indexes. Gene flow among these populations appears to be still enough to compensate for the effects of drift to maintain the levels of genetic diversity, and to avoid inbreeding and the accumulation of relatedness, irrespective of population size. This scenario seemed to occur in those populations that are located <103–145 km from the geographical centre of the whole population or <13–19 km to the nearest population (for *F*_IS_ and *R* respectively). Remarkably, this value is in fair accordance with the maximum dispersal distance of 20 km recorded for the Dupont's lark (Vogeli et al. 2010). Similarly, we also identified patch sizes where genetic indexes are not affected by an increase in isolation. Approximately 300 ha are needed to maintain inbreeding and relatedness values irrespective of the level of isolation, a size which match the patch size below which Dupont's lark densities increase as a response to patch contraction (Vogeli et al. 2010). However, larger patch sizes are required for genetic diversity (above 1800 ha) to maintain levels independent of isolation.

### Conservation implications

Some guidelines for Dupont's lark conservation can be extracted from the results of this study, especially regarding specific recommendations for minimum patch sizes and distances between populations needed to prevent genetic erosion. In terms of isolation, the distance to the nearest Dupont's lark population should not exceed 13 km. Populations separated by larger distances would lose territories and may additionally present a crowding effect with numerous potentially deleterious consequences. In this sense, we have detected a critical distance threshold of 30 km above which inbreeding and differentiation would increase dramatically. We also identified a minimum patch area of approximately 300 ha to avoid accumulation of inbreeding and relatedness, whereas larger patch sizes (>1800 ha, or 19 male territories) should be targeted to prevent the loss of genetic diversity. Although these thresholds are relative to our power to detect changes in genetic parameters, which is a function of sampling size, genetic markers and the time since fragmentation started, these results raise concern over the situation of the species. Attending to this study, the majority of Spanish populations are currently below these threshold sizes; approximately 90% of the occupied patches are smaller than 1800 ha and half of them cover <300 ha (range 20–5000 ha). Regarding the isolation of populations, 73% of them are separated by more than 13 km and 54% by more than 19 km from the nearest population (range: 5.5–173 km). Consequently, many Dupont's lark populations may be prone to suffer an ‘extinction vortex’ and raise the possibility that genetic factors contributed to the decline in population's viability (Vogeli et al. 2011) and even to the recent extinctions of small and isolated populations (Tella et al. 2005; Vogeli et al. 2010).

### Advantages and practical implications of the methodological approach

**Figure 4:**
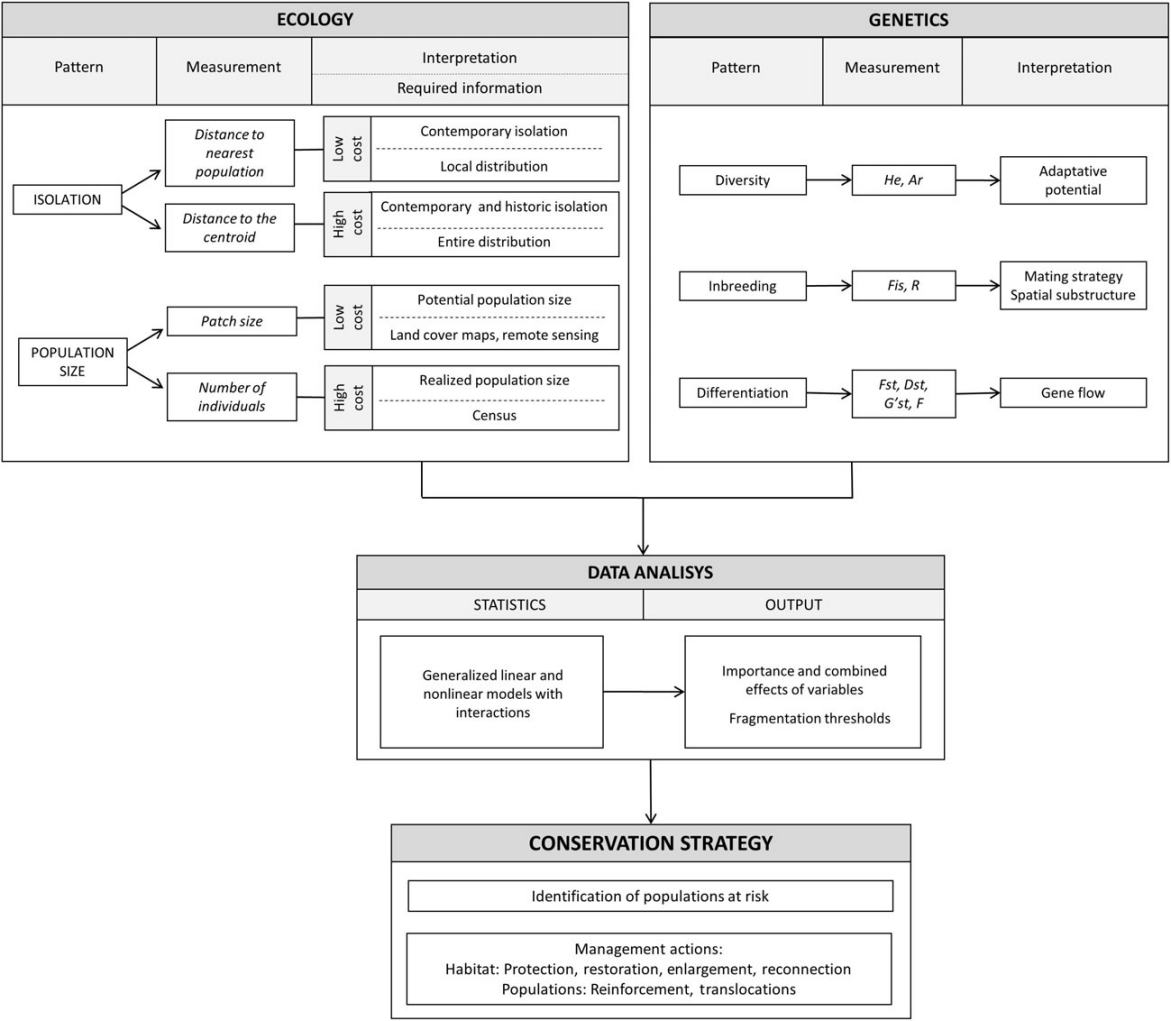
Overall framework of our methodological approach linking ecological and genetic data to conservation actions. The same approach could be applied to other study systems. In a first step (upper boxes), we show the concepts examined, the alternative variables used to measure them, as well as their interpretation, information required and time and economical cost of data collection, in the case of ecological data. The middle box shows statistical procedures and their expected outputs. The bottom box shows potential management actions derived from the results.

The combination of ecological and genetic data in a multivariate modelling framework (schematized in Fig. 4) allowed us to understand the effects and importance of different estimators of fragmentation on population genetics and to detect fragmentation thresholds from which the genetic health of local populations would become compromised. We assessed *a priori* defined hypotheses on the effects of habitat fragmentation on genetic erosion and tested them through a model selection approach. Those hypotheses involved alternative population and ecological parameters and the seldom considered interactions between them, an aspect that can help to improve management strategies. The strength of our approach for understanding the genetic processes derived from fragmentation was highlighted by the fact that finally selected models explained between 40% and 65% of the overall variance in genetic diversity, inbreeding and differentiation. Moreover, the use of alternative and complementary estimators of genetic diversity, inbreeding and differentiation (Fig. 4), which relate to different aspects of the genetic erosion processes, helped us to infer responses to fragmentation with different dynamics and at different spatio-temporal scales (Keyghobadi et al. 2005). This allows obtaining a broader picture of the various effects of habitat loss and fragmentation on the genetic composition of local populations. On the other hand, testing several ecological variables allowed us to contrast and compare their association with the genetic indexes and make more precise management recommendations. While previous work showed the need of increasing habitat size and connectivity to reverse the decline of this endangered species (Vogeli et al. 2010; Méndez et al. 2011), the fragmentation thresholds we are providing here offer practical guidance to wildlife managers.

Our conceptual and analytical framework (Fig. 4) can be broadened to other study systems involving any species threatened by habitat fragmentation. We used alternative variables to characterize population size and isolation, which are likely to differentially capture different aspects of the fragmentation process and that can vary between study systems. Number of male territories and patch size are alternative proxies of population size for our secretive study species, but they differ considerably in their estimation costs. Estimating the number of territorial males required costly and time-consuming censuses, while patch size is an easier and cheaper measure that can be obtained from remote-sensing or general-purpose land cover maps (Fernández 2013). However, actual population sizes, and even the proportion of breeding individuals, can be more easily obtained for more conspicuous species showing population fragmentation (e.g. Tella et al. 2013).

Distance to the centroid of all populations and distance to the nearest population can be considered as complementary measures of isolation. The former one has a ‘time since isolation’ component under our model of centripetal contraction, which makes it biologically more relevant in a context of recent fragmentation. However, it requires the knowledge of the distribution of all extant local populations, a kind of information that may not be available for all case studies. In this case, the use of simple and easy-to-measure, but still informative, variables (as distance to the nearest population) should still help to improve management actions.

Finally, our conceptual and methodological approach can be implemented at different spatial scales. Our study system covered the whole European distribution of an endangered species, but the same approach should work well for studies conducted at regional or metapopulation scales. We thus encourage the use of our approach (Fig. 4) to design optimal strategies for preventing further genetic erosion and for reverting it in species threatened by recent and/or ongoing fragmentation. A further step to improve this approach would be to incorporate the explicit dynamics of habitat fragmentation and population size with time estimates for the onset of isolation and contraction, a type of information that was unfortunately not available for our study system.

## Data archiving statement

Raw data used to generate the main results of this paper are provided in the manuscript.

## Supporting Information

**Figure S1.** Predicted relationships and partial contributions of population and ecological variables on genetic indexes: patch size *(area)*, population size as number of male territories *(N.pop)*, distance to the centroid of all Spanish populations *(D.centre)* and distance to the nearest population *(D.near)*. **Table S1.** Ranges and coefficients of variation of genetic indexes for all populations. **Table S2.** Evaluation of alternative models for genetic parameters based on population size and isolation variables. **Table S3.** Fitted generalized linear models genetic diversity, inbreeding, relatedness and differentiation.
