# Supplementary material for: Joint effects of population size and isolation on genetic erosion in fragmented populations: finding fragmentation thresholds for management
Source: Evol Appl. 2014 Mar 20;7(4):506–18. doi: 10.1111/eva.12154 (PMC4001448; doi:10.1111/eva.12154)

Ranges and coefficients of variation of genetic indexes for all populations. *N*= males captured; *H_e_*= expected heterozygosity; *F_IS_*= inbreeding; *D_ST_*= genetic differentiation; *F*= accumulated inbreeding; *Area*= patch size (Ha); *N.pop*= male territories; *D.near*= distance to the nearest population (Km); *D.centre*= distance to the centroid of all populations (Km).

|  | N | *H_e_* | *F_IS_* | *D_ST_* | F | Area | N.pop | D.near | D.centre |
| --- | --- | --- | --- | --- | --- | --- | --- | --- | --- |
| Min | 2 | 0.339 | -0.176 | 0.071 | 0.021 | 20 | 4 | 5.633 | 23.300 |
| Max | 61 | 0.638 | 0.290 | 0.196 | 0.170 | 5000 | 227 | 173.876 | 446.300 |
| Mean | 15.333 | 0.567 | 0.067 | 0.116 | 0.057 | 625.393 | 44.606 | 35.735 | 165.088 |
| sd | 15.152 | 0.064 | 0.0856 | 0.032 | 0.036 | 945.701 | 52.554 | 37.585 | 107.887 |

Evaluation of alternative models for genetic parameters based on population size and isolation variables. Genetic parameters were classified in diversity (*H_e_* = expected heterozygosity; *A_r_* = allelic richness), inbreeding (*F_IS_*), relatedness (*R*) and differentiation (*F_ST_*, *D_ST_*, *G’_ST_*, *F*). Model selection was repeated for two alternative measures of population isolation: distance to the centroid of all populations (*D.centre*) and distance to the nearest population (*D.near*). *N.pop* = population size, *Area* = patch size. Additive (+) and interactive (*) effects of isolation and population or patch size were considered. For each model , the corrected Akaike Information criterion (*AICc*) is reported, along with the difference with the best model (*∆AIC_c_*) and the relative weight of evidence for each model (*w_i_*) that can be interpreted as the probability of model *i* being the best model for the observed data, given the candidate set of models (two equally good models when *w_i_* < 0.5). Statistics of the most parsimonious model for each index are highlighted in bold.

|  |  | Models with *D.centre* | | | | |  | Models with *D.near* | | | | |
| --- | --- | --- | --- | --- | --- | --- | --- | --- | --- | --- | --- | --- |
|  |  | *N.pop* | *Area* | *D.centre* | *Area* +  *D.centre* | *Area* *  *D.centre* |  | *N.pop* | *Area* | *D.near* | *Area* + *D.near* | *Area* * *D.near* |
| *H_e_* | *AIC_c_* | -91.9 | -85.9 | -99.6 | -103.6 | **-115.6** |  | **-91.9** | -85.9 | -87.3 | -89.8 | -89.1 |
|  | *∆AIC_c_* | 23.8 | 29.7 | 16.0 | 12.0 | **0.00** |  | **0.00** | 5.9 | 4.5 | 2.1 | 2.7 |
|  | *w_i_* | 0.00 | 0.00 | 0.00 | 0.00 | **1.00** |  | **0.57** | 0.03 | 0.06 | 0.20 | 0.15 |
| *A_r_* | *AIC_c_* | -33.4 | -32.5 | -39.0 | -38.1 | **-56.0** |  | **-33.4** | -32.5 | **-33.7** | -32.9 | -32.6 |
|  | *∆AIC_c_* | 22.6 | 23.5 | 17.0 | 17.8 | **0.00** |  | **0.36** | 1.2 | **0.0** | 0.9 | 1.1 |
|  | *w_i_* | 0.00 | 0.00 | 0.00 | 0.00 | **1.00** |  | **0.23** | 0.15 | **0.28** | 0.18 | 0.16 |
| *F_IS_* | *AIC_c_* | -63.2 | -64.2 | -63.0 | -61.8 | **-79.0** |  | -63.2 | -64.2 | -63.2 | -62.1 | **-69.2** |
|  | *∆AIC_c_* | 15.7 | 14.8 | 16.0 | 17.2 | **0.00** |  | 6.0 | 5.0 | 6.0 | 7.1 | **0.0** |
|  | *w_i_* | 0.00 | 0.00 | 0.00 | 0.00 | **1.00** |  | 0.04 | 0.07 | 0.04 | 0.02 | **0.83** |
| *R* | *AIC_c_* | -63.2 | -64.6 | -66.5 | -65.2 | **-69.2** |  | -63.2 | **-64.6** | -63.9 | -62.8 | **-65.4** |
|  | *∆AIC_c_* | 6.0 | 4.6 | 2.7 | 3.96 | **0.00** |  | 2.2 | **0.8** | 1.5 | 2.6 | **0.0** |
|  | *w_i_* | 0.03 | 0.07 | 0.17 | 0.09 | **0.65** |  | 0.12 | **0.24** | 0.17 | 0.09 | **0.36** |
| *F_ST_* | *AIC_c_* | -137.9 | -135.5 | **-146.5** | -144.6 | -143.1 |  | **-137.9** | -135.5 | **-138.5** | -136.4 | -134.0 |
|  | *∆AIC_c_* | 8.6 | 11.0 | **0.00** | 1.91 | 3.4 |  | **0.6** | 3.0 | **0.0** | 2.1 | 4.5 |
|  | *w_i_* | 0.01 | 0.00 | **0.63** | 0.24 | 0.11 |  | **0.31** | 0.09 | **0.41** | 0.14 | 0.04 |
| *D_ST_* | *AIC_c_* | -133.2 | -127.4 | **-145.8** | -143.4 | -141.1 |  | **-133.6** | -127.4 | **-133.4** | -131.0 | -129.0 |
|  | *∆AIC_c_* | 12.6 | 18.5 | **0.00** | 2.41 | 4.7 |  | **0.2** | 6.0 | **0.0** | 2.4 | 4.4 |
|  | *w_i_* | 0.00 | 0.00 | **0.72** | 0.21 | 0.07 |  | **0.39** | 0.02 | **0.42** | 0.12 | 0.04 |
| *G’_ST_* | *AIC_c_* | -111.4 | -117.0 | **-135.1** | -132.7 | -130.3 |  | **-123.4** | -117.1 | **-122.7** | -120.3 | -118.3 |
|  | *∆AIC_c_* | 11.7 | 18.1 | **0.00** | 2.42 | 4.9 |  | **0.00** | 6.3 | **0.7** | 3.1 | 5.1 |
|  | *w_i_* | 0.00 | 0.00 | **0.72** | 0.22 | 0.06 |  | **0.48** | 0.02 | **0.34** | 0.10 | 0.03 |
| *F* | *AIC_c_* | -123.0 | -119.5 | **-139.5** | -137.1 | -134.6 |  | **-123.0** | -119.5 | -121.9 | -120.0 | -117.0 |
|  | *∆AIC_c_* | 9.5 | 20.0 | **0.00** | 2.42 | 4.9 |  | **0.0** | 10.5 | 8.1 | 10.5 | 13.0 |
|  | *w_i_* | 0.00 | 0.00 | **0.72** | 0.22 | 0.06 |  | **0.97** | 0.05 | 0.01 | 0.00 | 0.00 |

Fitted generalized linear models genetic diversity, inbreeding, relatedness and differentiation. Model coefficients are shown separately for hypotheses involving distance to the centroid of all populations (D.centre) and distance to the nearest population (D.near), respectively. *Interc.* = intercept. Significant coefficient estimates: * *P*<0.05; ** *P*<0.001.

|  | Final Models with D.centre | | | | | |  | Final Models with D.near | | | | | |
| --- | --- | --- | --- | --- | --- | --- | --- | --- | --- | --- | --- | --- | --- |
|  | R^2^ Adj |  | *Interc.* | Area | D.centre | Area : D.centre |  | R^2^ Adj | *Interc.* | Area | D.near | Area : D.near | N.pop |
| *H_e_* | 0.653 | Estimate | 0,775** | -0,024* | -0,001** | 0,000** |  | 0.225 | 0.472** |  |  |  | 0.029* |
|  |  | Std.Error | 0,065 | 0,011 | 0,000 | 0,000 |  |  | 0.031 |  |  |  | 0.009 |
| *A_r_* | 0,551 | Estimate | 3,137** | -0,101** | -0,000** | 0,000** |  | 0.040 | 2.840** |  | -0.040 |  |  |
|  |  | Std.Error | 0,161 | 0,027 | 0,000 | 0,000 |  |  | 0.262 |  | 0.026 |  |  |
|  |  | Estimate (2) |  |  |  |  |  | 0.030 | 2.339** |  |  |  | 0.031 |
|  |  | Std.Error (2) |  |  |  |  |  |  | 0.075 |  |  |  | 0.022 |
| *F_IS_* | 0,417 | Estimate | 0,495** | -0,072** | -0,003** | 0,000** |  | 0,215 | 2.300* | -0.365* | -0.228* | 0.037* |  |
|  |  | Std.Error | 0,114 | 0,019 | 0,000 | 0,000 |  |  | 0.719 | 0.120 | 0.071 | 0.011 |  |
| *F* | 0,436 | Estimate | 0,019* |  | 0,000** |  |  | 0.248 | 0.113** |  |  |  | -0.017* |
|  |  | Std.Error | 0,008 |  | 0,000 |  |  |  | 0.017 |  |  |  | 0.005 |
| *R* | 0,237 | Estimate | 0.350 | -0,057 | -0,002* | 0,000* |  | 0.137 | 1.700 | -0.296* | -0.177* | 0.031* |  |
|  |  | Std.Error | 0.181 | 0.028 | 0.001 | 0.000 |  |  | 0.848 | 0.137 | 0.085 | 0.013 |  |
| *D_ST_* | 0.410 | Estimate | 0.038** |  | 0.007** |  |  | 0.136 | 0.155** |  |  |  | -0.012* |
|  |  | Std.Error | 0.000 |  | 0.000 |  |  |  | 0.017 |  |  |  | 0.005 |
|  |  | Estimate (2) |  |  |  |  |  | 0.141 | -0.028 |  | 0.014* |  |  |
|  |  | Std.Error (2) |  |  |  |  |  |  | 0.058 |  | 0.006 |  |  |
| *Fst* | 0.249 | Estimate | 5709.06 |  | 181.88* |  |  | 0.769 | -323964** |  | 35754** |  |  |
|  |  | Std.Error | 10476.69 |  | 53.37 |  |  |  | 34798 |  | 3445 |  |  |
|  |  | Estimate (2) |  |  |  |  |  | -0.029 | 41645 |  |  |  | -1824 |
|  |  | Std.Error (2) |  |  |  |  |  |  | 21042 |  |  |  | 6163 |
| *Gst* | 0.403 | Estimate | 0.095** |  | 0.000** |  |  | 0.130 | -0.030 |  | 0.016* |  |  |
|  |  | Std.Error | 0.000 |  | 0.000 |  |  |  | 0.068 |  | 0.006 |  |  |
|  |  | Estimate (2) |  |  |  |  |  | 0.148 | 0.179** |  |  |  | -0.014* |
|  |  | Std.Error (2) |  |  |  |  |  |  | 0.019 |  |  |  | 0.005 |


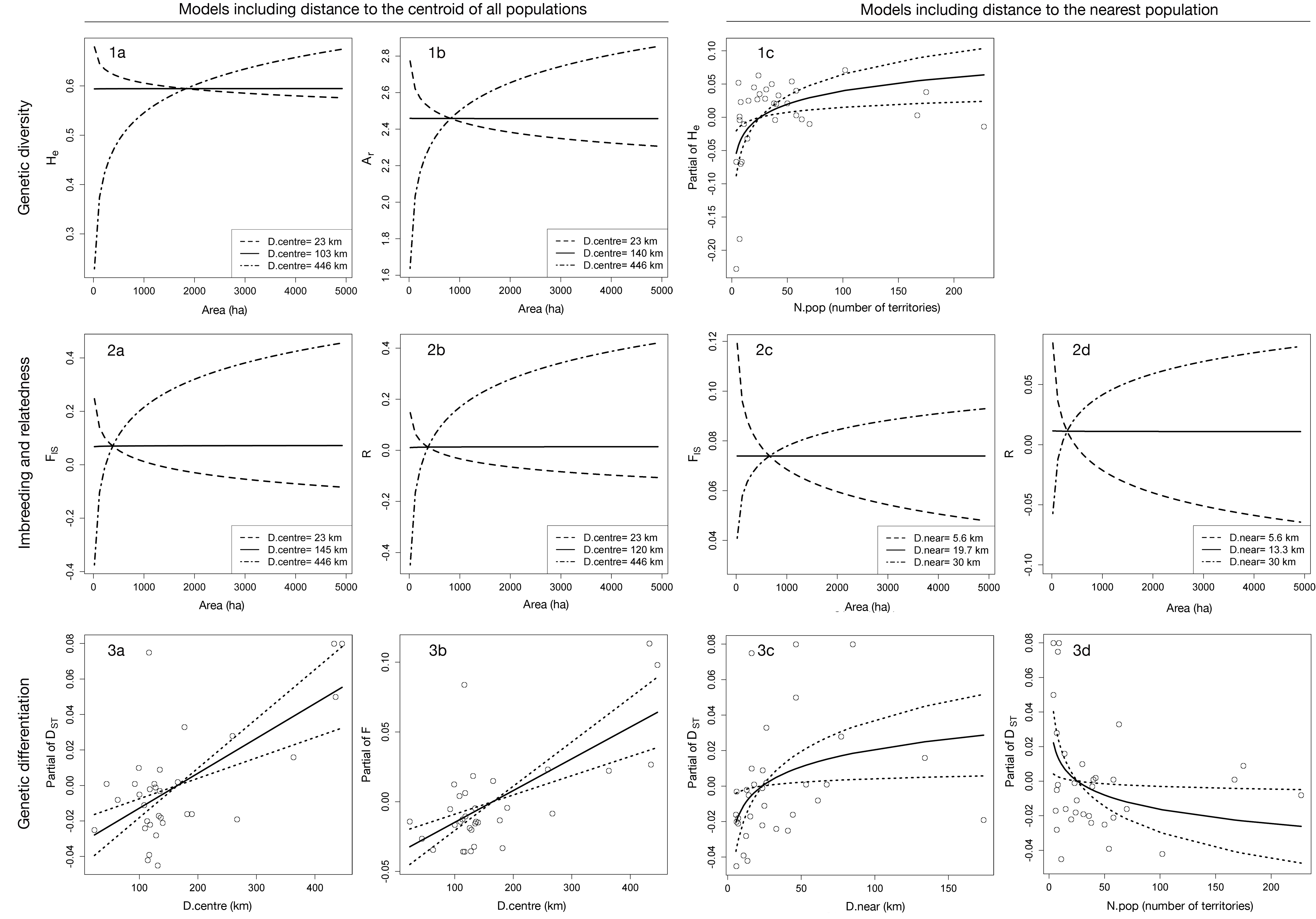

Supplement: Supplementary file 1 — Figure S1. Predicted relationships and partial contributions of population and ecological variables on genetic indexes: patch size (area), population size as number of male territories (N.pop), distance to the centroid of all Spanish populations (D.centre) and distance to the nearest population (D.near). Table S1. Ranges and coefficients of variation of genetic indexes for all populations. Table S2. Evaluation of alternative models for genetic parameters based on population size and isolation variables. Table S3. Fitted generalized linear models genetic diversity, inbreeding, relatedness and differentiation. [file eva0007-0506-sd1.doc]
